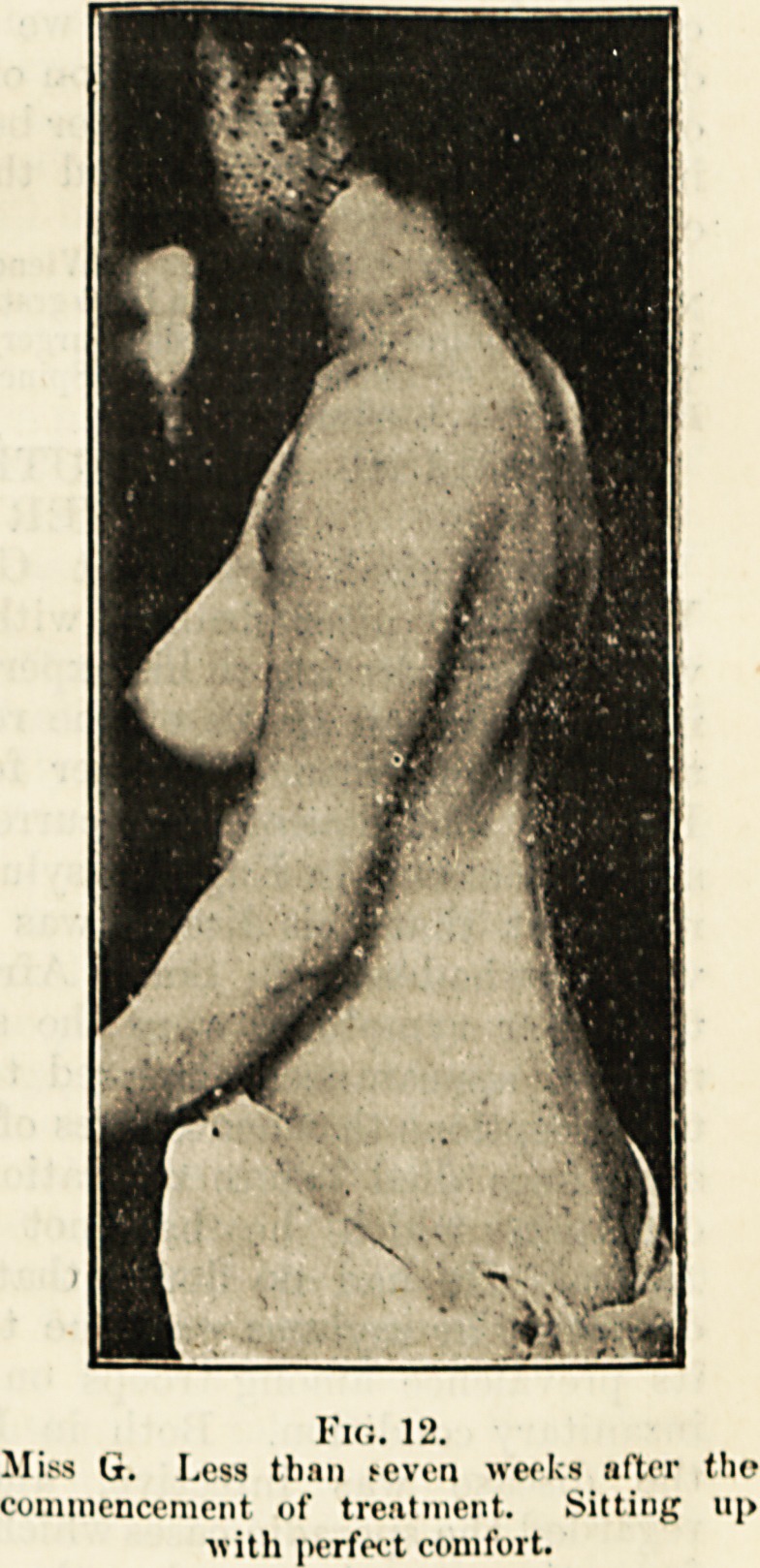# The Management of Lateral Curvature of the Spine (Scoliosis)

**Published:** 1901-10-05

**Authors:** E. Noble Smith

**Affiliations:** Senior Surgeon to City Orthopædic Hospital, London


### Hospital Clinics and Medical Progress.

### THE MANAGEMENT OF LATERAL CURVATURE OF THE SPINE (SCOLIOSIS).

By E. Noble Smith, F.R.C.S.Ed., L.R.C.P.Lond., Senior Surgeon to City Orthopædic Hospital, London.

One might almost say that lateral curvature of the spine is a symptom rather than a disease, for it is the result of a great many different conditions. Yet when the deformity is once established the affection assumes, whatever may be its cause, so definite a character that it fairly claims the position of a distinct disorder. In dealing with lateral curvature, however, we must consider from a practical point of view the various steps which have led up to the deformity as we first see it, and we must not expect to be able to apply a specific kind of treatment which will meet the requirements of all cases alike.

In the present day there seems to be almost as great an inclination on the part of medical men to establish, as there is on the part of the public to accept, "systems" of treatment for various kinds of disease. Thus we have the *light* treatment of lupus, the *open-air* treatment of phthisis, the *serum* treatment of diphtheria, and various other special methods of dealing with special morbid conditions. Every scientific medical man knows that such particular lines of treatment have a limited value, and that it is to the judicious combination or selection of various remedies that he must look for the best results in any particular class of cases. This being so, it stands to reason that in dealing with scoliosis, a disease which, as already stated, arises from a variety of causes, no special "system" of treatment is applicable. Yet we find the "exercise" treatment or the "mechanical" treatment considered by certain persons as quite opposed the one to the other; while in truth we may combine these and other methods, or use each separately as may seem required. These statements may seem to the reader but the record of very simple facts or truisms, and yet they must be emphasised, because lateral curvature has been subjected to special systems of treatment perhaps more than any other surgical affection. These systems of treatment have been to a great extent founded upon theories of causation. The general view at the present day is that the fault lies in the muscular system, and this fault is usually described as "muscular weakness," but no one has attempted to explain why the muscles of the back alone should be specially attacked in this way. A theory once very prevalent was that curvature was the result of unequal action of the muscles on the two sides of the spine. It was Delpech who, in the first half of the last century, advocated this theory very strongly, but Werner,[1] in 1851, "demonstrated," as Bauer maintains, that the "muscular antagonism" theory was a gross error.

Lorinser[2] took quite another view, and advocated an old theory that the deformity originated in a "softening, general infiltration, and osteoporosis of the vertebral bodies." In these and other conflicting views we see attempts to evolve "systems," and it is probably fair to state that nearly all these observers were right and all were wrong. In other words, we may well believe that scoliosis depends upon a variety of causes. Even the extravagant view of Guérin that the deformity was caused by muscular contraction cannot be quite set aside (as it was by a committee of the "Académie des Sciences" in Paris in 1844), for do we not see lateral curvature produced by contraction of the sterno-mastoid muscle in torticollis, and from contraction of the adductors of a thigh? Such instances as these would, however, be described as *exceptional* by theoretical writers, but there are so many exceptions that they embrace almost the whole range of cases. We have inequality in length of legs; inequality in weight from loss of an arm; forced positions in trades and school work; contraction of a side from pleurisy, and many other special conditions. These, it may be said, are exciting causes, but they are so potent in their effects that they may produce deformity even when no predisposition exists. It is the predisposition, however, which most demands our consideration, for if we can effectually remove the tendency then the counteracting of the exciting influences becomes comparatively easy. With regard to this aspect of the subject, probably no more enlightened views have been expressed than those of Buehring.[3] Translated by Bauer,[4] Buehring attributes the chief predisposing cause to defective vitality, especially in girls at the age of puberty, "a low state of hæmatosis . . . anæmia . . . inefficient nutrition of the various structures of the body, depriving bones and cartilages of their usual firmness and elasticity and rendering them susceptible to an alteration of their respective forms." . . . "The softness of bone is therefore the simple result of a low state of nutrition, and not of any specific structural disease, as, for instance, rachitis, osteomalacia, osteitis, etc. Nor is the softness so great as to be affected by the weight of the body alone, though sufficient to give the spine an unusual degree of flexibility." My own experience with regard to a large number of cases is absolutely in accordance with these views of Buehring. Undoubtedly muscular weakness may exist as a result of such deficient vitality; but we must not lose sight of the primary cause nor of the necessity of dealing with the weakened basis of support, the skeleton, while we are attempting to develop the strength of the active agents in movement—the muscles.

*Management.*—The treatment of lateral curvature must first be directed to the removal of all exciting causes. If the length of the legs is unequal the spine will be curved in the lumbar region towards the side of the shorter limb. This inequality must be corrected. Up to one third of an inch the amount of shortness of one limb may be corrected by the addition of the deficiency to the heel only of the boot, but if it exceeds that amount something should be added to the sole also. For instance, if one inch is the amount of shortening, then it is well, besides adding that extra thickness to the heel, to put one third of an inch on the sole, otherwise the toes will be too much depressed; but the thickness on the heel alone corrects the shortness. If the deficiency is greater, say, 2 or 3 inches, then a carefully constructed platform for the foot is required. The best raised boot of this kind is that made by the O'Connor Extension Company, and it is so cleverly constructed that the shortness is disguised. If one leg is fixed in an adducted position, then, although it be the same length as the other, yet the patient will have to lift up the pelvis on the side of the affected leg until the foot is flat above the ground. It will then be seen that the foot does not reach the ground, but requires (so long as the forced adduction remains) artificial

Fig. 1. Fig. 2.

Fig. 1.—Diagrammatic representation of a leg held in an adducted position by ankylosis or by contraction of adductor muscles.
Fig. 2.—Diagrammatic representation of the effect produced by the patient bringing the adducted leg parallel with the sound leg. The pelvis is tilted, the spine curved to the left, and the affected limb held some distance above the ground.

support to enable the patient to walk; the spine, moreover, will be curved in the opposite direction.

It is always desirable to correct this condition either by division of the adductors, if their contraction is the cause, or in the case of fibrous ankylosis, by forced movement, or, if there is bony union, by osteotomy. If the deformity has not lasted long, the straightening of the limb will alone suffice to cure the spine; but if the adducted limb has existed long enough to cause structural changes in the spine, then some special means must also be taken to correct the curvature. In infantile paralysis of one leg there is often lateral curvature from a giving way of the affected side, and then the proper support of the leg and the necessary operations will in the same way relieve the crooked spine. Congenital displacement of one hip is another cause of inequality, but in these cases the spine is generally curved in the opposite direction from the efforts of the patient to lift up the limb of the affected side. These instances will suffice to show the line of treatment to be adopted when the base of the spine is thrown out of its proper plane. In those cases of lateral curvature in which, when the patient stands upright, the pelvis remains level, we may yet have an inequality as an exciting cause. The habitual posture of the patient may be one-sided, and the postures engendered by school work are proverbially bad. This part of the subject I have dealt with extensively in other writings, and, therefore, I will not enlarge upon it here, except to state that very few people engaged in teaching have given much attention to the subject. At a few schools, however, the principals have, at my suggestion, provided means for pupils who are delicate to do their work lying in the prone position, this being the only certain way of preventing bad postures during school work. The prone posture, if provision be made for the patient's legs to slope downwards from the hips, with the body lying entirely on the flat part of the couch, allows the spine to rest in its best position. Almost every body-movement of the patient, when resting in this position, brings into action the muscles of the back. In reading, for instance, the pupil is obliged to hold her head up by using the dorsal muscles. The supine position in recumbency is very inferior to the prone. When supine, the pupil must rest perfectly quiescent, and with a small pillow under the lumbar part of the spine. Every movement towards an upright posture, such as is necessitated by reading a book held in the hands, tends to round the back. It brings into action the abdominal and pectoral muscles, and leaves the dorsal muscles unused.

The use of a properly constructed prone couch, or cushions placed in the same position on the floor, will alone do a great deal to benefit a weak or slightly-curved spine. If a patient suffering from lateral curvature could always occupy such a position, and could also carry out a suitable course of exercises at several periods during the day, she would be following out a very effective plan of treatment. But consider the drawbacks! What a terribly irksome life for any young girl to lead to be constantly lying recumbent! Such treatment would have to be carried out for many months to produce permanent good to the spine, and therefore it cannot be recommended. Moreover, other more simple and equally effective means are at our disposal. However well devised may be our plan of exercises (even if while these exercises are being carried out the spine can be brought into a straight position), there must be a temporary relapse of the lax spinal joints into their former crooked position in the intervals between

Fig. 3.—Patient lying on the prone couch. The sloping part is from the hips downwards.

the exercises. When a patient is actively employed, as in playing out-door games, or even in walking about, there is far less tendency for the spine to relapse than when she is sitting in a chair, and therefore we may adopt the middle course of only using the couch for resting, and prohibiting the use of chairs. Exercises for lateral curvature require much care and consideration. There seems to be a very general idea not only that lateral curvature is one special kind of weakness, but that for that weakness there is but one special remedy, and that remedy "Exercises," however carried out. Consequently, any professor of gymnastics or calisthenics or Swedish, Danish, or any other movement cure is thought competent to carry out the necessary treatment. A good proof of the fallacy of these views is the fact that of several hundreds of very severe and quite incurable cases of lateral curvature which have come before me, the great majority have previously gone through a course of special exercises during some period of the development of the affection, and such treatment has failed in their cases to arrest the deformity. To be effectual, the exercises must be very carefully devised and intelligently carried out, and the knowledge of the surgeon as to

Fig. 4.—Dorso-lumbar curvature, the curve being most prominent about the tenth dorsal vertebra.

the exact nature of the affection and the physiology as well as the anatomy of the parts involved must be applied in supervising the work. I have elsewhere described (*Lancet*, July 7th, 1900) the plan upon which I have carried out such a course of treatment. Some of the movements there given are not new, but the most important one, that which counteracts rotation, was devised by me many years ago, and has proved

Fig. 5.—The same patient as in Fig. 4 drawing back the elastic expander with the right arm, thus using the muscles between the shoulder and the spinous processes of the deflected dorsal vertebræ, straightening the whole spine and un-rotating the vertebræ. The vertical lines from the outer side of the pelvis show the relative positions of the side of the body, without and with the active exercise.

most effectual in influencing this troublesome part of the affection. I here show the *modus operandi* of this exercise. Here the patient is sitting in a chair, but in [practice she would be recumbent, either in the prone or supine position.

However perfect the exercises may be, however efficiently and persistently they may be carried out, they yet may fail to cure or even to prevent the increase of deformity. The effect of such treatment must be closely watched, and directly it is seen that the remedy is not alone effective, mechanical help must be substituted or added. I know too well how strong is the prejudice against mechanical apparatus, and such prejudice is founded upon facts. From time almost immemorial monstrous machines have been made by mechanicians for crooked spines, and it remained for my late colleague at the City Orthopædic Hospital, Mr. E. J. Chance, to devise an apparatus constructed upon scientific surgical principles. Is it complained that a spinal instrument interferes

Fig. 6.—Shows depression of the chest and roundness of the back in the felt jacket.

with the development of muscles? The splint I refer to has the very contrary effect: it encourages muscular action. Is it said that the development of the body is retarded? The apparatus I mention promotes it. The chest is left free for expansion; the arms are drawn back, but left free for up and down movement, and there are no crutches. While wearing this instrument, the patient, when he can hold himself straight for a short time, is free from support, but directly he relaxes his position he is checked from falling into bad postures by the apparatus. It is not so much a support as it is a guide to the patient to hold himself straight. It therefore encourages movement in the right direction. Without any apparatus, the patient affected with lateral curvature cannot as a rule move his arms or body in ordinary actions without bending his spine into worse positions; but with the apparatus I refer to he is obliged to move himself in the best directions.

The worst kind of apparatus is the felt jacket; it binds up the body in a fixed stay, impervious to moisture, and can only prevent stooping by pressing on the front of the thorax. The illustrations fig. 6 and 7 show the comparative effects of the two appliances.

The girl here shown, fig. 6, had worn the felt jacket for two years, and her spine was getting worse. It will be seen how the chest is hollowed out and the head depressed. The other splint, fig. 7, was put on, and the photograph taken directly after the felt jacket had been removed, thus showing the immediate effect of the more scientifically-devised machine. I have elsewhere so fully described these points of difference, and expressed my views upon the treatment of lateral curvature,[5] that I will write no more, except to say that, as far as exercises alone will do good, by all means let us apply them; but when this system fails, or when we have to deal with a decided change in the shape of the spine, we ought to supplement the treatment by the use of mechanical apparatus, for by that means alone are we able to effect a cure or an improvement.

Fig. 7.—Shows the effect rapidly produced by the "Adaptable Metal Splint" in the same patient as fig. 9.

I will now describe the principles upon which I have acted in treating lateral curvature of the spine. General physical weakness, often associated with too rapid growth, being a very common cause, it happens that when a child has outgrown its strength there are plenty of exciting causes in the ordinary postures of life which will have a directing influence in producing a particular form of curvature.

General weakness leads to inability on the part of the patient to sustain himself or herself in an upright position, and especially is this so when sitting down without the back being properly supported. In the majority of these cases one might almost infer that an undue softness of bone and laxity of ligament would accompany a general condition of weakness, but we have also direct evidence that this softness and laxity are present in many instances.

We may find other joints of the body besides those of the spine affected by this sort of weakness, exemplified by flat-footedness and weakness of knees, and particularly a great flexibility of the finger-joints and wrists.

If nothing be done for this class of patient, we generally see a rapid increase in the curves, and the deformity quickly develops. Children who have outgrown their strength, and are perhaps two or three inches taller than they ought to be for their age, and whose vitality is below its normal condition from insufficient nitrogenous food, are those who chiefly suffer.

The knowledge that some undue softness of bone has to do with the production of lateral curvature was apparent, as I have already stated, to many of the observers of a past period, and it is probable that this condition consists in nothing more than want of vitality in development.

Everything tends in the present day to the increase of nervous energy at the expense of the physical, and it seems possible that such a tendency shows itself in the rapid growth of the descendants of men and women whose nervous systems have been so excessively developed.

If we accept this view as to the general condition of the majority of patients who develop lateral curvature, we shall modify very considerably the popular ideas as to necessary treatment. It having been determined by some medical practitioners that the muscular system alone is weak, it has been thought that it is a rational remedy to resort to systematic exercises to restore these structures to a state of health, and doubtless in the treatment as time goes on active muscular exercises form a useful part of our remedial measures. I would, however, urge that not only is such a method incapable by itself to meet all the difficulties of these cases, but in a great many instances it will do more harm than good. The muscular system cannot be treated beneficially by itself, and so long as the bony and ligamentous tissues remain soft and liable to easy alteration in their form, so long will undue exercise tend to produce further deformity rather than relieve it.

A weak, enfeebled, overgrown child will bear very little exercise with benefit, and will often be strengthened rather by temporary rest than by an increase in its movements. In contrast to these delicate children one meets with typically robust young people who are suffering from lateral curvature, and there may even be very severe deformity in these cases. It is difficult in all instances to trace the origin of such a condition. It may have commenced when this particular patient was in a weak state like those already described; but sometimes we see a direct cause in the form of a short leg or in some of the other exciting causes already mentioned. As to treatment of the weakly cases, which undoubtedly form the great majority of these deformed patients, surgeons who have had to deal largely with them have from time immemorial felt the necessity of sometimes using mechanical apparatus, but nearly all of them have contented themselves with leaving the exact design of this apparatus to the ingenuity of the instrument-maker.

Those few surgeons who have endeavoured to design surgical supports for the spine have not been very successful in their attempts. The consequence has been that these surgical appliances have been constructed upon purely mechanical knowledge, and little note has been taken of the physiological needs of the patient. In nearly all the appliances that have been made in the past the principle adopted has been that of a rigid corset, by which it is attempted to prop up the body with crutches under the arms, or to press upon the thorax from the front. The interference with the muscular development which such an apparatus brings about has led in recent years to strong opposition to the use of spinal apparatus, and many surgeons have a great prejudice against them. At one time I shared this prejudice, but that was before I knew much about the subject. When I came to study the difficulties of dealing with lateral curvature, I found that if we were to produce any alteration in the shape of a deformed spine we must in the majority of cases use some mechanical apparatus. At that time no description of a mechanical appliance which did not interfere with the development of the muscles had appeared, but an instrument was being used at the City Orthopædic Hospital, devised by Mr. E. J. Chance, which possessed many of the qualities which were necessary *to prevent the spine from falling into bad positions, whilst it allowed ample opportunity for the body to develop and the muscles to act.* I have to a certain extent modified this apparatus while retaining its principles. It is not an instrument-maker's machine fitted closely to the body, but is a very simple apparatus affording a means by which the surgeon can bring pressure or support to bear upon any part of the trunk he likes. The principles upon which it is constructed are as follows.

The first object is to keep the spine in as upright a position as possible in an antero-posterior direction, for, as it is a well-known fact that flexion (stooping) increases the deformity, and especially increases the rotation of the vertebræ, on the other hand, extension (uprightness) has a contrary effect. To further this object, patients are often made to lie down, or rest in chairs which fit into the lumbar region, and so support the spine in a good position. Instruments usually fail to a great extent in this respect because they depend upon crutches for propping up the body, and crutches would have to be very high and painful, as well as detrimental in other ways, if they kept the spine sufficiently upright. In weak backs the tendency is for the back to bulge posteriorly in the lower dorsal and lumbar regions, and it is certainly desirable to control this projection. Instruments, as usually made, either leave this part unsupported or they curve into the hollow and act as permanent supports. The tendency of the latter machines is to relieve the muscles of the back from action, and to conduce to their degeneration and ultimate weakness.

To overcome this evil effect I discarded rigid stays and restraining spinal instruments (including felt and other jackets) and used the light support above referred to.

In slight cases it will be observed that when the patient stands erect the back is straight, or nearly so, and the natural lumbar curve is formed. When, however, the patient sits down, the spine immediately bows backwards and the lateral curve takes place. If we can prevent this bowing backwards we retard the increase of, or remove, the lateral curve.

The simplest form of the apparatus is shown in fig. 9, in which it is applied to a case of slight lateral curvature connected with drooping of the shoulders and head. Fig. 8 shows the patient before treatment. Fig. 9 shows the result of a few weeks' use of the apparatus. The supporting bar, made of light steel, reaches from the level of the shoulder, A, to the seat, B. The soft shoulder-straps act directly backwards. C is the square pad placed in the lumbar region, which prevents the back from protruding posteriorly. The pelvic belt retains the lower part of the apparatus in position. The

Fig. 8. Fig. 9.

belt, D, restrains the abdomen from coming too far forward. This band is never made very tight, but the back is balanced between it and the pad at C.

In sitting (when the back ought to be rested) the body rests against the pad, C. In standing or walking the body ceases to rest against C, but is restrained from coming forwards by D.

There is no interference with any of the muscles of the back, or of any other muscles, but a support is always ready to restrain the body from falling into a bad posture when the muscles cease from acting. In most cases side pads are attached to the central bar, A B. The pad, C, is so arranged that it does not act when the patient is holding himself erect—when, in fact, it is not wanted—and so the dorsal muscles are allowed to act; but directly the spine is allowed to subside, then the back comes in contact with the pad, and is prevented from bulging any further. Some cases, no doubt, may be cured without mechanical means, but even such will require far more time and attention and trouble to the patient than if they are treated with this apparatus, and, moreover, the results of the non-mechanical treatment are never certain and often fail. I have stated this fact in other words, as follows :—"Those few cases which would be permanently benefited by the movement cure, will derive an equal amount of good from the treatment here advocated at a tenth part of the trouble and expense."

In the case shown, fig. 8, the usual projection backwards of the lumbar or dorso-lumbar region does not occur, but generally the latter part gives way in a very marked degree in the sitting posture. This is well shown in the following illustration, fig. 10.

As the case proceeds the pad, C, is gradually brought nearer to the natural position of the spine, and thus a back which at first protruded very considerably is brought by degrees into a natural position. Never, however, is the pad placed so forward that it retains the spine in one position ; on the contrary, there is always room for action of the muscles and movement of the vertebral joints. The result of this plan of treatment is that patients with backs so weak that they cannot sit upright for more than a moment will, in a very short time—say, a month, or in even less time—show a very decided increase in power. Backs which have been quite flabby to the touch have become hard and muscular in a few months. The apparatus has to be put on the first thing in the morning, and not taken off until the patient retires to bed. Such an apparatus does not lead a patient to depend entirely upon its use ; that is to say, at any time in the process of treatment the support may be thrown aside, and the patient be, to some extent, the better for having used it, or if used, as it should be, until the patient's back has become quite straight and strong, it may be thrown aside without any period of gradual leaving off. Considerable care and discretion have to be exercised in adjusting the pads, for if we attempt to improve the position too much at first, it will hurt the patient, and, in fact, be intolerable. I find it of the utmost importance in the treatment of this affection to avoid producing pain or discomfort, and when a case is not proceeded with too rapidly the result invariably is that all disagreeable symptoms, such as aching or pain, are very soon relieved. The back pad is retained in its place in a very simple manner, and when it is in use—*i.e.*, when the back rests against it—it is supported, first, by the shoulder straps already described, which tend to draw the arms back and develop the chest ; secondly (at lowest part) by a simple pelvic belt. None of the straps need be buckled very tightly.

In the less severe cases this apparatus, in its more simple form, will enable us to effect a cure, but when the lateral curving is very marked, something more must be done ; the lateral plates must be applied to counteract the lateral curves. Here, at least, it may be thought, the principle of allowing freedom to the muscles must be abandoned, but it is not so. The lateral plates, if properly adjusted, will take effect, while the muscles of the back are allowed to act freely. I always advise the patient to endeavour to "get away" from the lateral pressure. If patients hold themselves so that the pressure is relieved, they are effecting *by muscular action* more than the statical pressure of the plates can effect, whilst when not making this exertion the plates support the back from falling into a worse position.

It is this muscular action which removes the objection sometimes raised against lateral plates, that they cannot affect the spine very much, because they have to act (in the dorsal region) through the ribs. And even when we have to act upon the ribs, in the majority of cases, the pressure is well

Fig. 10.
Case of Miss G. Upon her first visit. She was quite unable to sit up straighter, except for a moment, and with a great effort.

Fig. 11.

Miss G. Wearing the apparatus.

Fig. 12.

Miss G. Less than seven weeks after the commencement of treatment. Sitting up with perfect comfort.

transferred to the spine itself. The practical results of this treatment confirm the theoretical, although it must not be supposed that good results can be obtained very easily. Mechanical aptitude and considerable mechanical knowledge are absolutely necessary to the surgeon who would straighten a curved spine. It is unreasonable to expect success if we delegate to instrument makers the devising, the adaptation, and the adjustment of instruments with which we aim at solving a very difficult surgical problem. Knowledge of the physiology of the movements of the parts affected, of the anatomy, and the nature of the pathological changes which may take place, is required before the operator can form a judgment as to how best to apply and adjust his mechanical measures. The use of this apparatus is one of the most important helps to the treatment of lateral curvature, but in addition we have, of course, to deal with the general condition of weakness, and one of the most potent remedies for benefiting the patient in this respect is massage and the carefully-devised exercises already referred to.

[1] Reform der Orthopædie. [2] Wiener Med. Wochenschrift, No. 22, 23, 24. [3] Die Siebliche Rückgrats-Verk-rümmung, Berlin, 1851. [4] Lectures on Orthopædic Surgery, by Louis Bauer, New York, 1868. [5] "Curvatures of the Spine." 4th Edition. Smith, Elder, and Co., London.

## Figures and Tables

**Fig. 1. Fig. 2. f1:**
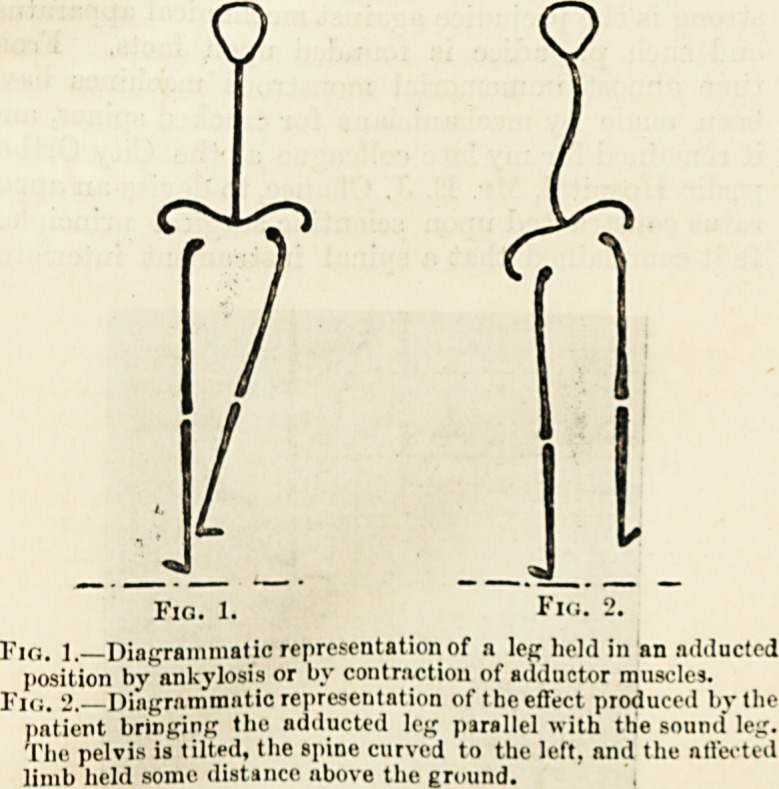


**Fig. 3. f2:**
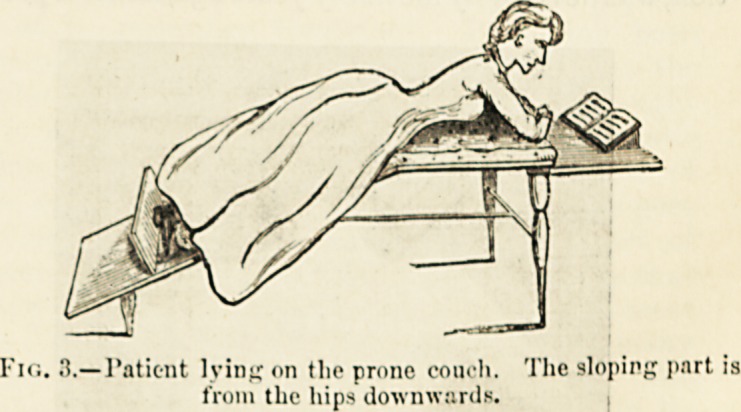


**Fig. 4. f3:**
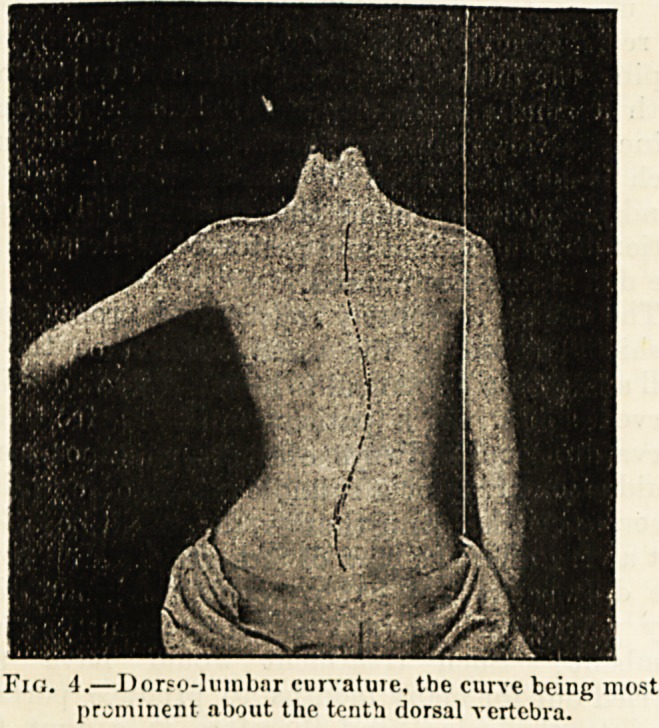


**Fig. 5. f4:**
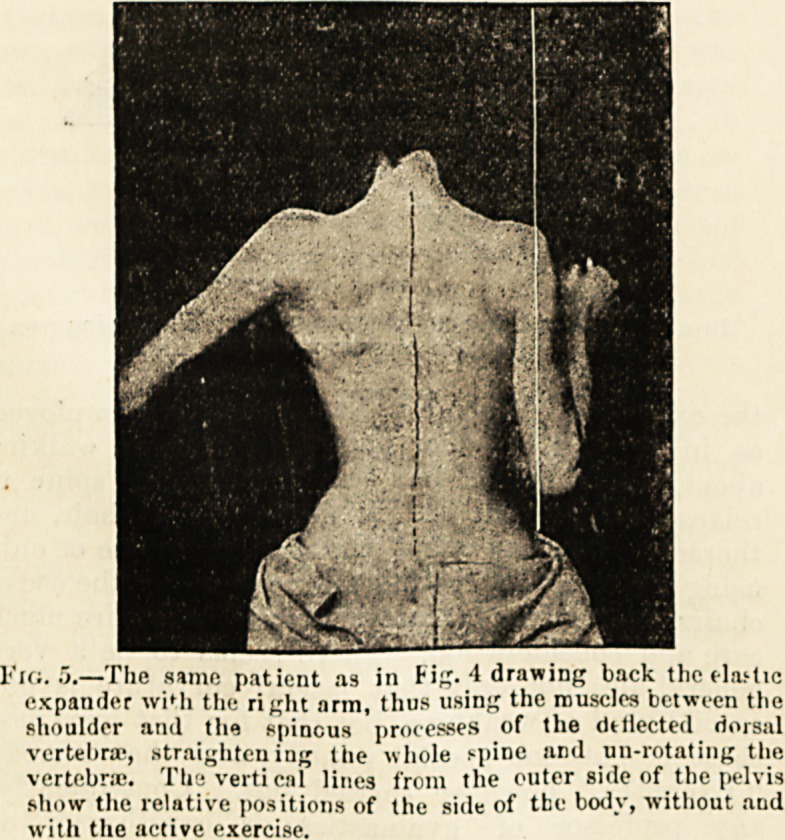


**Fig. 6. f5:**
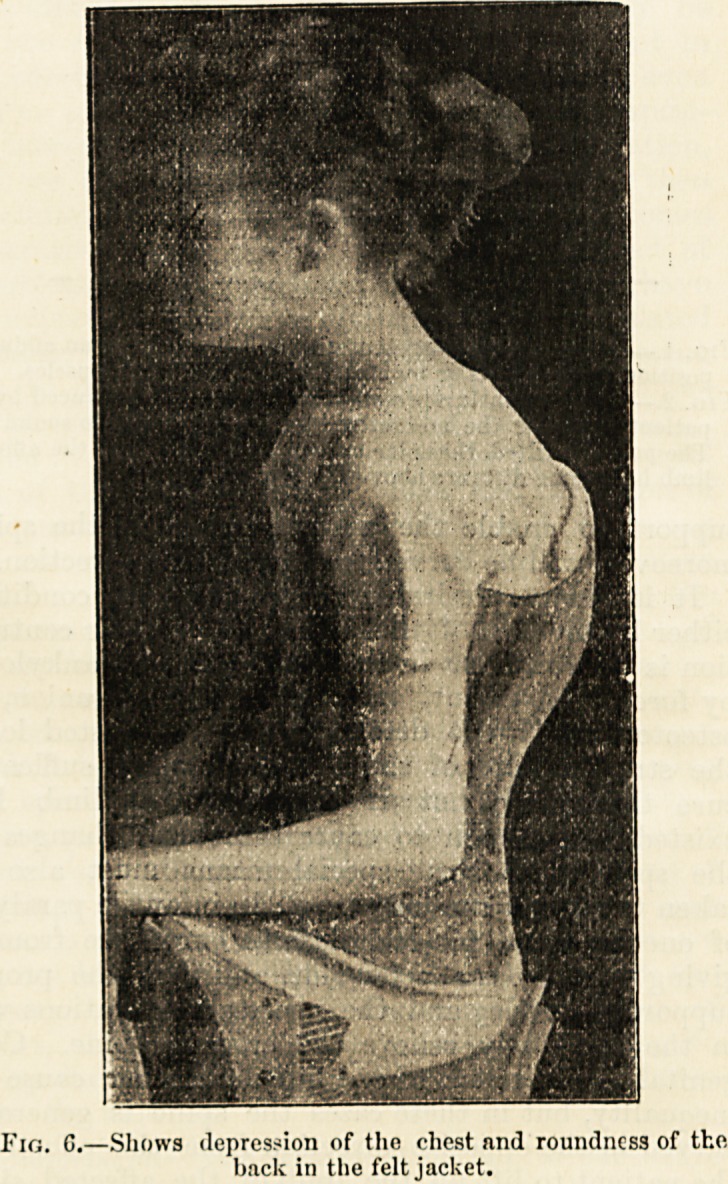


**Fig. 7. f6:**
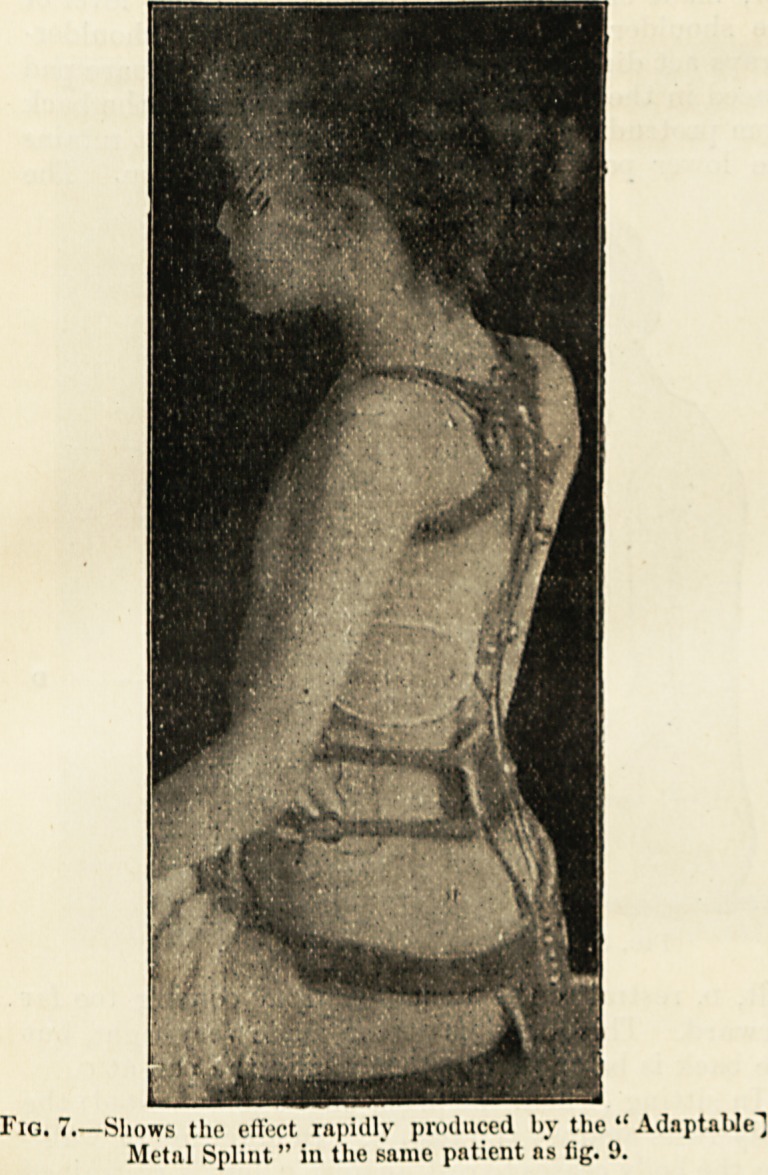


**Fig. 8. f7:**
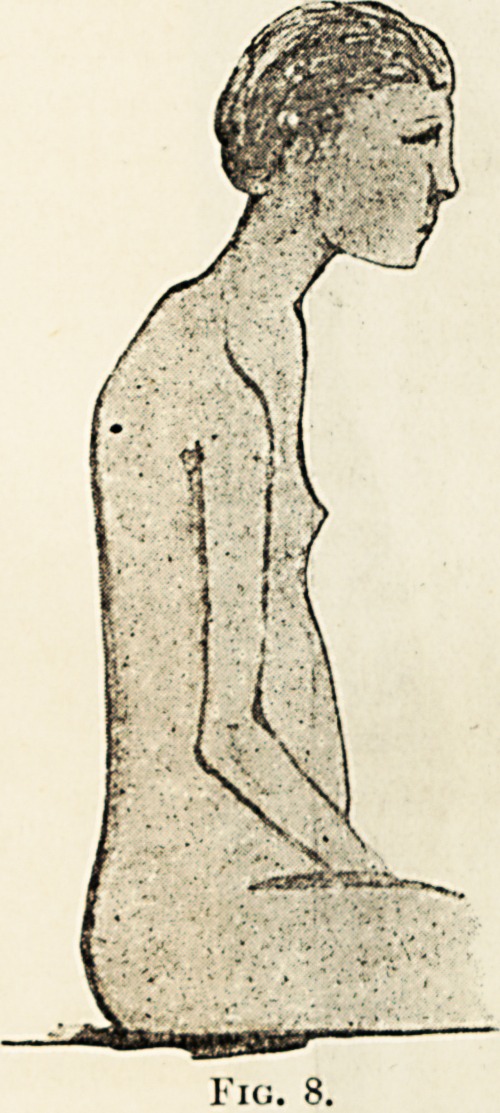


**Fig. 9. f8:**
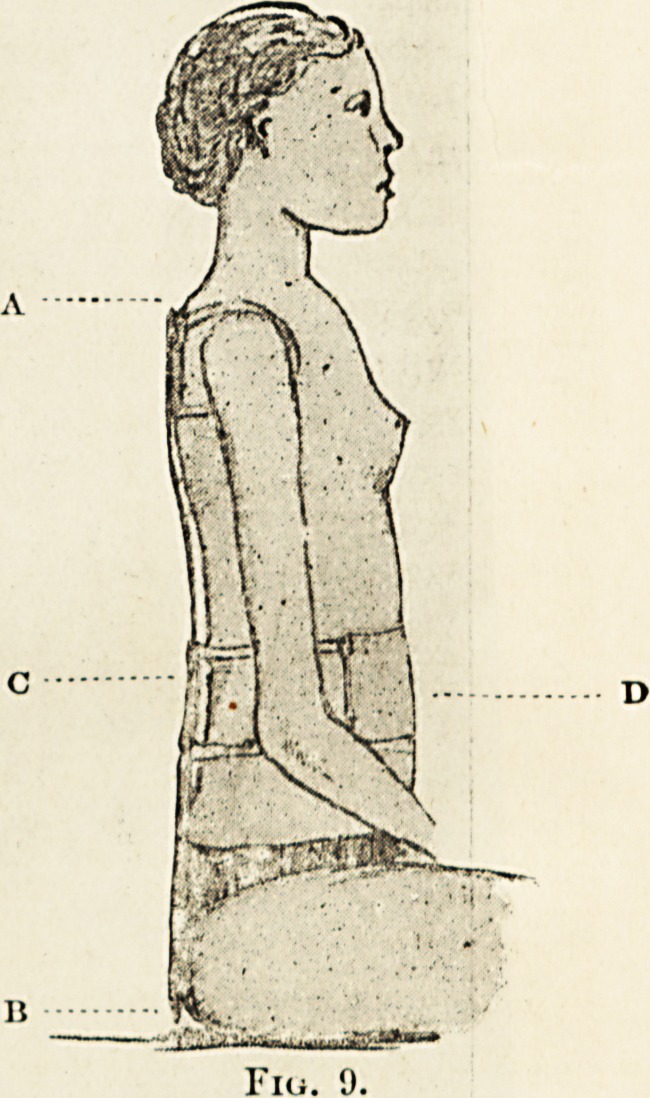


**Fig. 10. f9:**
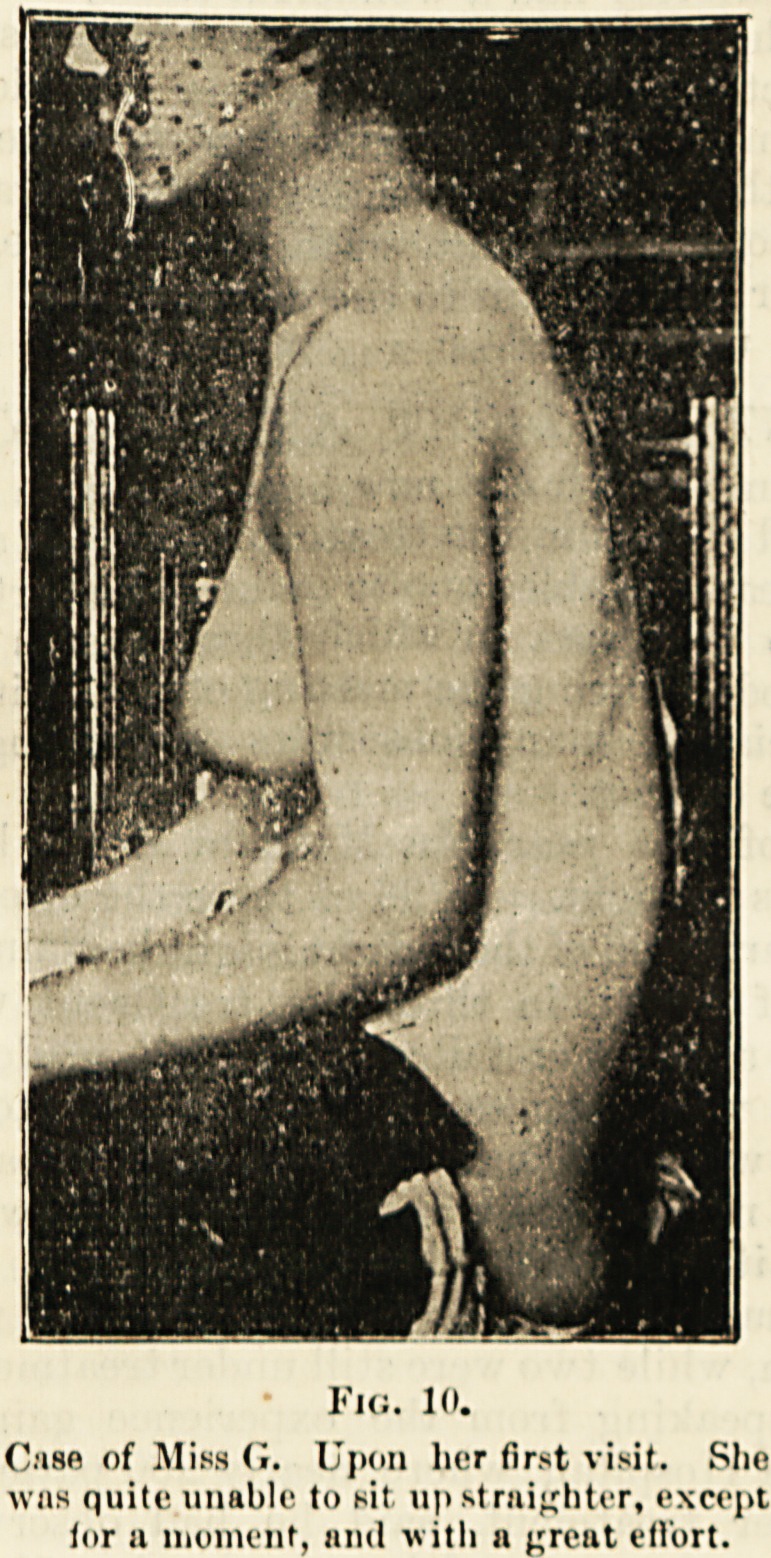


**Fig. 11. f10:**
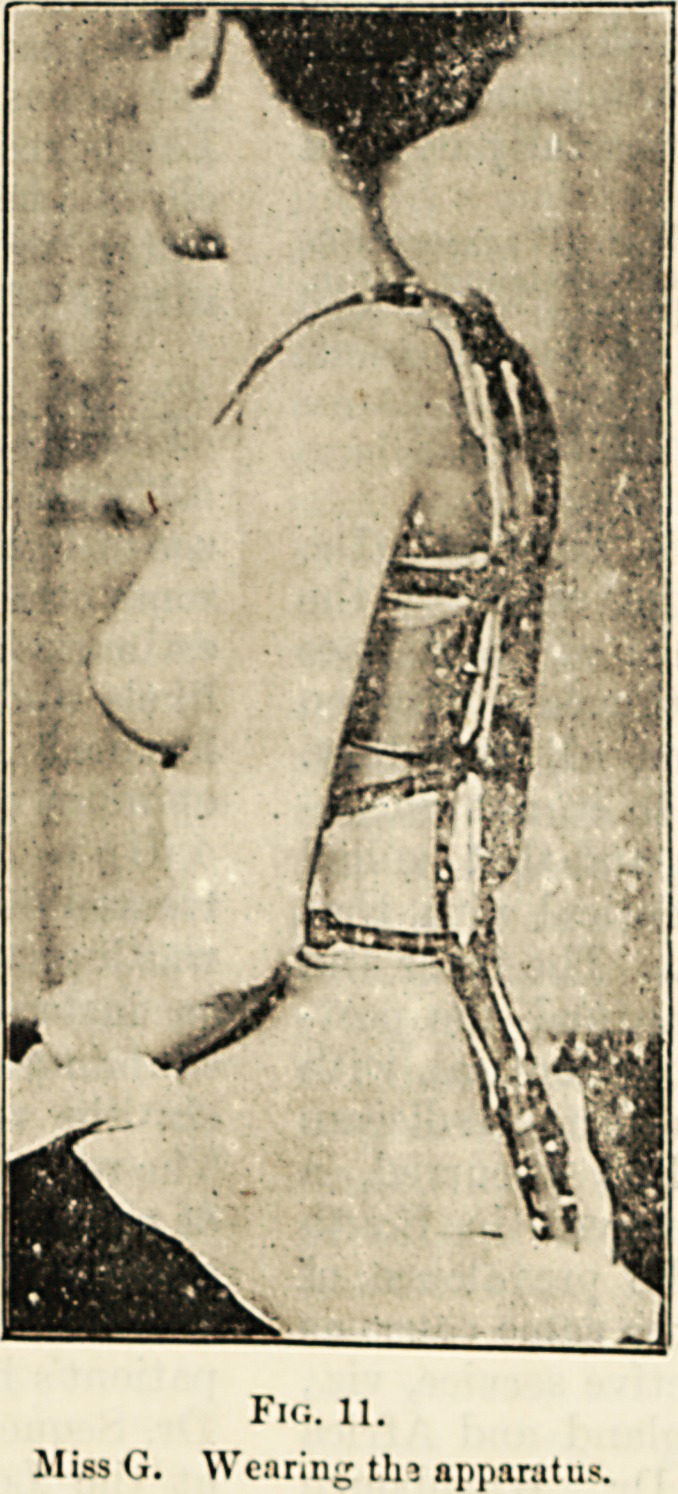


**Fig. 12. f11:**